# PREDICTIONS OF RISK TOLERANCE ACROSS DISEASE SCENARIOS AMONG OLDER ADULTS

**DOI:** 10.1093/geroni/igz038.3111

**Published:** 2019-11-08

**Authors:** Catherine Ju, Brian Carpenter

**Affiliations:** Washington University in St. Louis, St. Louis, Missouri, United States

## Abstract

Advance care planning requires older adults to contemplate what kind of medical care they would prefer if faced with serious illness. Those decisions involve weighing risks of medical treatments with quality of life. The purpose of this project was to explore older adults’ tolerance for risk in medical treatment scenarios. Thirty-three adults over age 60 (mean age = 74.9, SD = 8.9) were presented with four medical scenarios reflecting increasingly severe diseases (urinary incontinence, kidney failure, dysphagia, Alzheimer disease). For each, they were asked whether they would accept a treatment with increasing risk of death (5%, 50%, 95%). Participants also recorded demographic characteristics and completed a physical and mental health measure. Older adults were more willing to accept hypothetical treatments with lower risk of death (Cochran’s Q = 27.8 - 39.0, p’s < .001), and they were more willing to accept treatments when faced with more severe diseases (Cochran’s Q = 8.7 - 29.8, p’s < .05). There were no significant associations between risk tolerance and demographic and health characteristics. These results suggest that older adults are diverse in their willingness to accept medical treatments risks, at least when presented with hypothetical medical scenarios. Medical professionals and family members who collaborate with older adults should be aware of this diversity of preferences and check assumptions based in demographic and health characteristics. Given these nuances, interventions to promote communication about medical preferences are essential to patient-centered advance care planning and medical decision making.

